# Performance of Tönnis triple osteotomy in older children with developmental dysplasia of the hip (DDH) assisted by a 3D printing navigation template

**DOI:** 10.1186/s12891-022-05669-z

**Published:** 2022-07-26

**Authors:** Fei Liu, Kai Tang, Peng-fei Zheng, Zhi-qun Zhang, Gang Ling, Yue Lou

**Affiliations:** Department of Orthopedics, Children’s Hospital of Nanjing Medical University, Nanjing, 210008 China

**Keywords:** 3D printing (3DP), Navigation template, Older children, Developmental dysplasia of the hip (DDH), Triple osteotomy

## Abstract

**Background:**

The objective of this study is to investigate the preparation of a navigation template via a computer-aided design (CAD) and 3D printing (3DP) in order to improve the effectiveness of Tönnis triple osteotomy in older children with developmental dysplasia of the hip (DDH).

**Method:**

Thirty-eight older children who received Tönnis triple osteotomy were included in this study. Among them, 20 were categorized as the 3DP navigation template group (3DP group), and the remaining 18 were categorized as the conventional surgery group (CS group). Data, including preoperative and postoperative pelvic sharp angle (SA), lateral center-edge angle (LCEA), acetabular roof angle (ARA), acetabular head index (AHI), crossover sign (COS), ischial spine sign (ISS), operation time (OT), intraoperative blood loss (IBL), and number of radiation exposures (NORE) were recorded for both groups. In addition, the therapeutic effect was evaluated at the last follow-up, according to the McKay criteria and Severin’s criteria.

**Results:**

In the 3DP and CS groups, the mean OT was 126.6 ± 17.6 min and 156.0 ± 18.6 min, respectively; the mean IBL was 115.0 ± 16.9 ml and 135.7 ± 26.5 ml, respectively; the NORE were 3.3 ± 0.8 times and 8.6 ± 1.3 times, respectively. There were significant differences in the OT, IBL, and NORE between the two groups (*P* = 0.03, 0.05, < 0.001, respectively). At the last follow-up, the 3DP and CS groups displayed SA of 41.8 ± 2.3° and 42.6 ± 3.1°, respectively; LCEA of 35.6 ± 4.2° and 37.1 ± 2.8°, respectively; ARA of 6.9 ± 1.8° and 9.8 ± 2.6°, respectively; and AHI of 86.6 ± 4.1% and 84.3 ± 2.8%, respectively; COS(+) of 5 hips and 4 hips, respectively; ISS(+) of 6 hips and 7 hips. We observed no statistical differences in the SA, LCEA, ARA, AHI, COS and ISS between the two groups (*P* = 0.918, 0.846, 0.643, 0.891, 0.841, 0.564, respectively). According to the McKay criteria, the 3DP group had 10 excellent, 6 good, and 4 general hips, whereas, the CS group had 12 excellent, 4 good, and 2 general hip. There was no statistical difference between the two groups (*P* = 0.698). In 3DP group the postoperative Severin’s grading included 13 hips in grade I, 4 in grade II, 3 in grade III. Alternately, in the CS group, the postoperative Severin’s grading included 11 hips in grade I, 5 in grade II, 2 in grade III. The Severin ‘s criteria also showed no statistical difference between the two groups (*P* = 0.945).

**Conclusions:**

Base on our analysis, our CAD-3DP-fabricated navigation template assisted Tönnis triple osteotomy in older DDH children, it reduced operation time and number of radiation exposures. However, no significant differences in radiological assessment and functional outcomes were observed when an experienced surgeon performs the surgery. Therefore, Surgeons who have less experience in triple osteotomy profit more from the application of this technology.

## Background

Developmental dysplasia of the hip (DDH) is one of the common lower limb deformities in pediatric orthopaedics. It has a high disability rate and seriously affects the physical and mental health of pediatric patients [1–3]. Children with developmental dislocation of the hip (DDH) require personalized treatment, based on their ages and pathological characteristics of the hip [4]. The therapeutic effect of acetabular dysplasia in childhood is relatively good. In case of older patients, Tönnis triple osteotomy is a viable option, with satisfactory therapeutic effect [5, 6]. In fact, its accurate direction of acetabular rotation can improve the head and acetabular alignment, thus avoiding the outward movement, as well as the under- or over-correction of the rotating acetabular center. Therefore, the treatment for DDH is to establish a concentric, congruent, stable and safe reduction of the hip. However, it remains difficult for surgeons to determine the degree of correction after acetabular osteotomy, so as to achieve ideal acetabular inclusion. Insufficient coverage of the anterolateral femoral head can lead to early instability, while excessive coverage tends to limit the range of motion, thus resulting in femoro-acetabular impingement, which can induce severe complications like residual acetabular dysplasia and osteoarthritis. In recent years, computer aided design (CAD) and 3D printing (3DP) are widely used in the preoperative planning stage of multiple disciplines, such as, orthopaedics [7, 8], neurosurgery [9], and oral and maxillofacial surgery [10]. Nevertheless, only a few studies have reported the results of CAD and 3DP assistance of Tönnis triple osteotomy, and demonstrated the clinical advantages of this new technology in comparison with those of conventional surgery. In this study, we provide objective information regarding the outcomes of Tönnis triple osteotomy using a navigation template and to compare it with that of a conventional surgery.

## Materials and method

### General information

We conducted a retrospective study of children with DDH admitted to our hospital from October 2016 to March 2019, all patients age 18 or younger who underwent Tönnis triple osteotomy were identified via an intradepartmental medical record search. A power analysis was performed before the study to justify this as a sufficient amount of patients for review. Their average age was 11.5 years old (range: 8.1–16.6 years old). There were eight males and thirty females. The left side was affected in eighteen patients, and the right side was affected in twenty. The pediatric patients were next divided into two groups: twenty cases were assigned to the 3DP navigation template group. Among them, four were males and sixteen were females. Additionally, nine patients were affected on the left side, and eleven on the right. The ages ranged from 8.7 to 16.6 years old (mean age: 11.7 ± 2.5 years old). The CT data of patients were collected prior to surgery. A CAD navigation template was printed using 3DP for intraoperative use. Meanwhile, eighteen cases were placed in the CS group. Among them, three were males and fifteen were females. In addition, nine patients were affected on the left side and nine on the right. The ages ranged from 8.1 ~ 14.8 years old (mean age: 11.5 ± 2.0 years old) (see Table 1). The inclusion criteria were as follows: (1) age ≥ 8 years old; (2) preoperative X-ray and CT examination confirmed DDH diagnosis; (3) unilateral DDH; (4) follow-up period of no less than 24 months. Contraindications to the osteotomy were poor joint congruency, and partial narrowing or disappearance of the joint space with the hip in abduction position. All patients underwent preoperative pelvic X-ray and CT images. The CS group was the same as the 3DP group. Both groups had the same rehabilitation programs. The recorded data included preoperative and postoperative SA, LCEA, ARA, AHI, COS, ISS，OT, IBL, and NORE for both groups. The pelvic supine antero-posterior radiographs were used to measure these parameters. The lateral osseous margin of the acetabular roof was used as an acetabular landmark to measure these parameters [11]. LCEA was measured using the pelvic longitudinal axis, defined as a line perpendicular to the line, connecting the center of both femoral heads [12]. OT was recorded from skin incision to skin closure via anesthesia documentation. Intraoperative blood loss was estimated withr colorimetric method. The therapeutic effect was evaluated at the last follow-up, according to the McKay criteria and Severin’s criteria. Information was provided, and a consent form was signed by each patient prior to the initiation of the study. All patient data were anonymous. All surgeries were performed by one experienced surgeon (Fei Liu) who had carried out over 100 conventional triple osteotomy and 30 navigation assisted surgery before this trial. We introduced the purpose of this study before surgery, then all patients were asked to choose whether to Tönnis triple osteotomy assisted by a 3D printing navigation template voluntarily. This study was approved by the Ethics Committee of the Children’s Hospital of Nanjing Medical University.

**Table 1:** General information of DDH children between 3DP group and CS group

Parameter	3DP group (*n* = 20)	CS group (*n* = 18)	*P* value
Age, y	11.7 ± 2.5	11.5 ± 2.0	0.628
Gender			
Male	4	3	0.396
Female	16	15	
Side			
Left	9	9	0.819
Right	11	9	
Follow-up time, M	30.3 ± 3.4	31.5 ± 2.5	0.726
sharp angle(°)	52.7 ± 4.8	48.2 ± 2.6	0.689
center-edge angle(°)	16.5 ± 2.8	14.7 ± 6.8	0.897
acetabular roof angle(°)	36.8 ± 2.6	37.4 ± 3.1	0.916
acetabular head index(°)	56.8 ± 2.1	49.8 ± 3.2	0.732
crossover sign			
positive	3	2	0.723
negative	17	16	
ischial spine sign			
positive	4	6	0.351
negative	16	12	

### Design and printing of individualized navigation templates

**Fig. 1:**
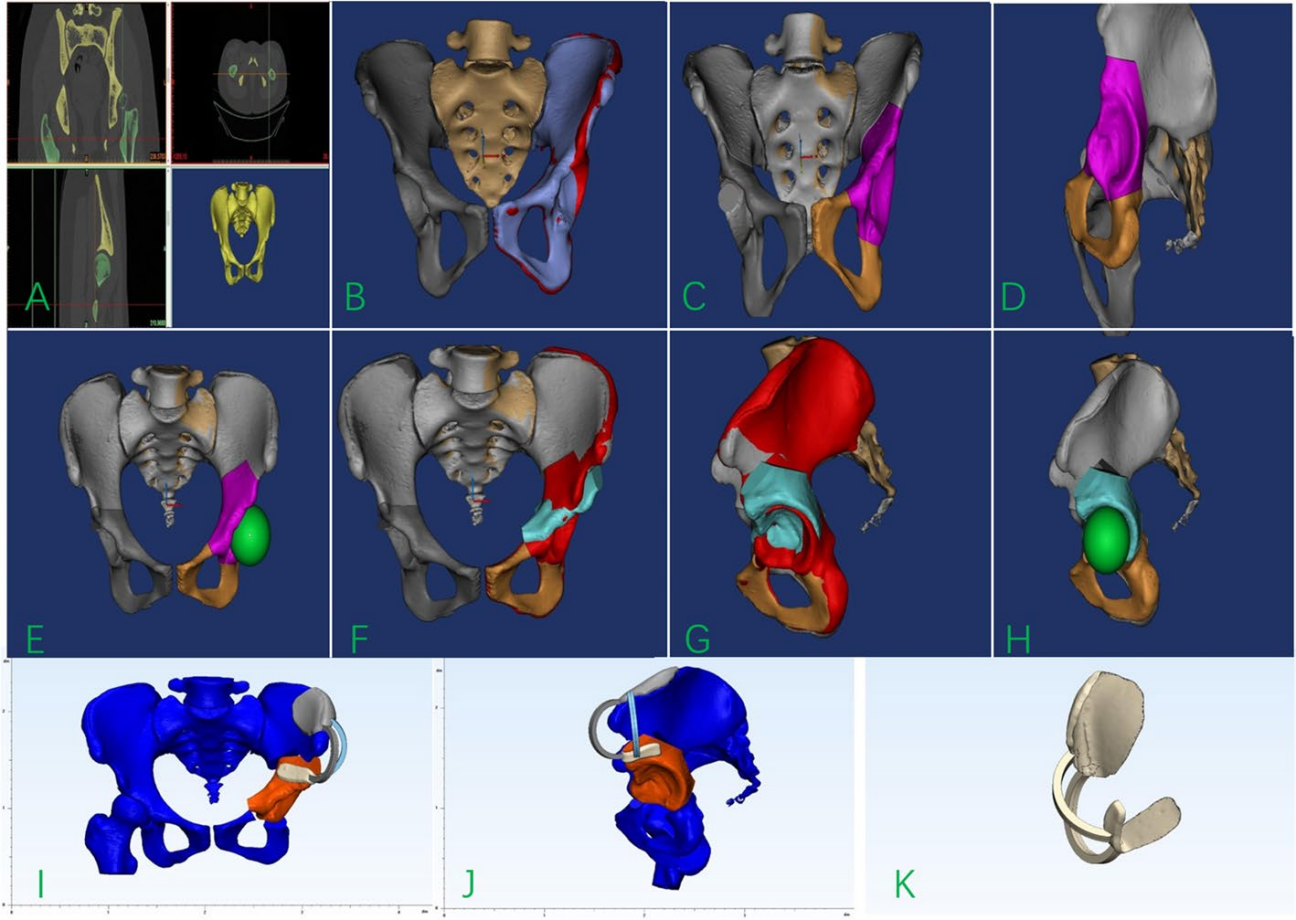
Design and application of the customized navigation template. **A** The pelvis data was imported into the Mimics software for 3D reconstruction. **B** The imaging of the unaffected hip was mirrored to the contralateral side with the positive view of the affected side. **C**, **D** The simulation of the Tönnis triple osteotomy. **E** The positive view of a perfect sphere was simulated as the femoral head. **F**-**H** the imaging of the unaffected acetabulum was rotated to allow it to coincide with the mirrored imaging as much as possible. **I**, **K** The navigation template, designed in 3-MATI and exported in STL format

The preoperative pelvis and proximal femur data were scanned using a 64-slice spiral CT (Philip, Netherlands). A spiral CT scan of the pelvis and femur was obtained using specifc scan parameters (120 kV, 150 mAs, 1-mm-thick slices, pixel matrix, 512 × 512). Original DICOM data were imported into Mimics 17.0 software (Materialise, Belgium) to generate a 3D reconstruction of the femur and pelvic (see Fig. 1A), Additionally, the model surface was smoothed. Anatomical characteristics of the unaffected acetabulum was used as the standard to evaluate the deformity of the affected acetabulum in the 3D reconstructed pelvic model. Appointing the median sagittal plane as the mirror surface, the imaging of the unaffected hip was mirrored to the contralateral side (see Fig. 1B). The simulated bone cutting plane of the superior pubic ramus and superior pubic ischium was simulated using the navigation template in the Mimics software (see Fig. 1C and D). Subsequently, a perfect sphere was simulated as the femoral head (see Fig. 1E), and this was taken as the center of rotation, and the acetabulum was rotated outward and downward to allow it to coincide with the imaging results of the healthy side as much as possible (see Fig. 1F-H). Next, the rotated osteotomy model was imported into 3-MATIC software, A Boolean operation was used to acquire the iliac crest surface morphology, and a 3-mm-thick process in the opposite direction was used to create a matching substrate, and the two bridge portion of the navigation template was used as the connecting substrate. Lastly, the 3D navigation template model was constructed. (see Fig. 1I- K). Once the navigation template model design was completed, the data were imported into the 3D printer (FusionTech, China) in the STL format, and the navigation template was printed out using medical PLA (polylactic acid) materials. Total model preparation time was approximately 8 hours. Materials cost was at most $15.

### Operation and postoperative treatment

Patients were anesthetized and placed in a supine position, The affected side was padded to form a 30° angle and sterilized. The CS group received single-incision extraperiosteal Tönnis triple osteotomy as follows: skin incisions were made beginning at the anterior-superior iliac spine, and continuing distally along the sartorius muscle. The fascia lata was carefully incised and the lateral femoral cutaneous nerve was separated and carefully retracted. The ilium is approached by subperiosteal dissection of the external and internal ilium muscles. The anterior joint capsule was exposed, and the tendon of the iliopsoas muscle resected from the lesser trochanter, then sciatic branch at the base of the acetabulum was exposured. Ischial osteotomy was performed using a 30-degree Ganz osteotome. The osteotomy position was 0.5 cm from the distal end of the incisura acetabuli. The ischial osteotomy is performed positioned at the level of the infracotyloid groove, just distal to the acetabulum. The osteotome is directed toward the lesser sciatic foramen. It was oblique from the anterior to the posterior, and passed upwards completely through the ischium, just below the acetabulum. Flexing and adducting the hip, the iliopsoas tendon was relaxed. The soft tissue was separated along the pubic branch while protecting the femoral artery and vein. While protecting the soft tissues with a Hohman, the pubis, including its thick overlying periosteum, was cut medial to the iliopectineal eminence to avoid injuring the joint and the triradiate cartilage. The region of the periosteum that was not cut by the Ganz osteotome at the pubic osteotomy site was then cut with dissecting scissors to ensure sufficient rotation at the pubic osteotomy site during acetabular rotation. A Schanz screw was inserted into the roof of the acetabulum prior to the iliac osteotomy to redirect the acetabulum. Following the osteotomy, the Schanz screw was connected to a T handle, which was then used to pull the acetabulum laterally and slightly anteriorly to cover the femoral head. We stabilized the iliac osteotomy with four stainless-steel Kirschner wires with a diameter of 2.5 or 3.0 mm. An intraoperative AP pelvic radiograph or image intensifier was used to assess the correction.

**Fig. 2:**
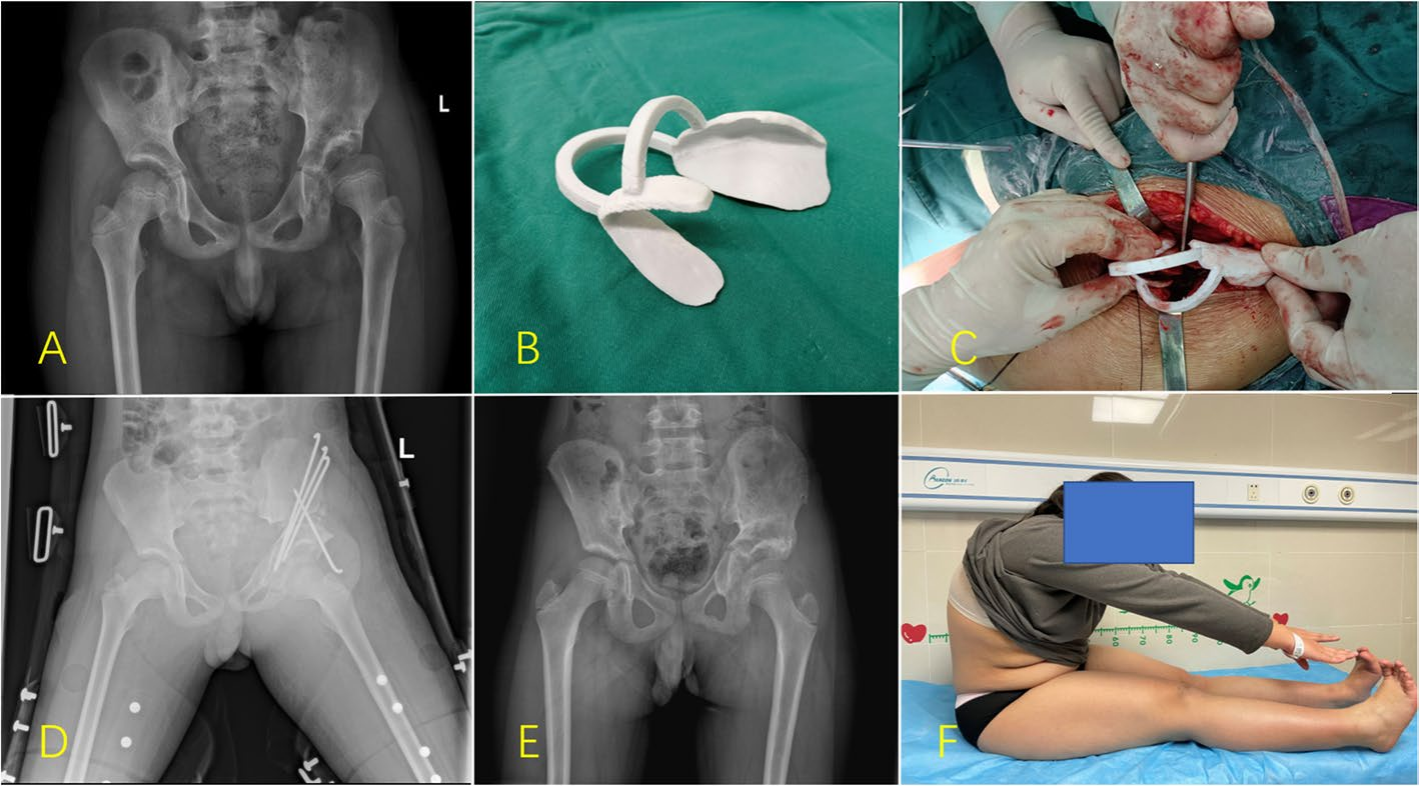
Tönnis triple pelvic osteotomy and follow-up **A** Anterior pelvic radiograph. **B** Intraoperative 3D navigation template. **C** Intraoperative diagram of the rotating acetabulum after sciatic, pubic, and iliac osteotomy. The rotation direction and angle were restricted with a navigation template. **D** Pelvic plain radiograph 1 day after surgery. **E** Pelvic plain radiographs 2 years after surgery. **F** Gross functional appearance at the last postoperative follow-up

The 3DP navigation template group received the same triple osteotomy procedure as the CS group. The 3D navigation template was used to perform the intraoperative acetabular rotation. Once the navigation templates matched well with the corresponding anatomical markers of the acetabular fragment, four Kirschner wires, with a diameter of 2.5 or 3.0 mm, were used to fix the osteotomy with Cross-pinning configuration. An intraoperative AP pelvic radiograph or image intensifier was used to assess the correction in the same manner as discussed above (see Fig. 2). The hip was then immobilized in a spica splint for 6 weeks. The OT, intraoperative blood loss (IBL) with colorimetric method, number of radiation exposures (NORE), and other related indexes were recorded.

### Clinical and radiographic evaluation

**Fig. 3:**
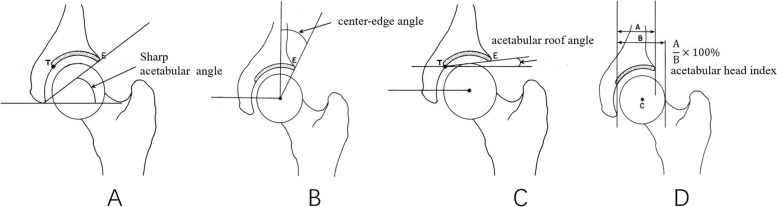
Common measurement index of older pediatric patients with hip dysplasia: sharp angle (**A**), lateral center-edge angle (**B**), acetabular roof angle (**C**), acetabular head index (**D**)

Three independent investigators not involved in the surgical procedures examined the patients at follow-up. Radiographic measurements were performed preoperatively and postoperatively by three radiologists and repeated after an interval of 2 weeks. Clinical follow up was performed using McKay criteria [13]. Radiographic analyses included preoperative and postoperative measurements of the sharp angle, lateral center-edge angle, acetabular roof angle, acetabular head index, crossover sign, ischial spine sign (see Fig. 3). The final radiographic outcome was evaluated on the anteroposterior pelvic radiographs, according to the Severin grading system [14]. Severin grades I and II were considered satisfactory outcomes, while Severin grades III and IV were considered unsatisfactory outcomes.

### Statistical analysis

We performed a post hoc power analysis using G*Power software (ver.3.1.9.7; Heinrich-Heine-Universität Düsseldorf, Düsseldorf, Germany), to estimate sample sizes required for 80% power and a type I error (α) of 0.05 to detect an effect size of 0.4 using both analysis of variance estimates at significance level 0.05. All measurement data were presented as the mean and standard deviation. The radiographic parameters of the acetabular position were compared using the paired t test. The categorical variables were compared with a chi-square test or Fisher’s exact test. Statistical significance was considered when the *P* value was less than 0.05. All statistical analyses were performed using the statistics package SPSS 23.0.

## Results

Navigation templates were successfully designed for all patients. All navigation templates matched the model exactly in simulated surgery. The intraoperative navigation template and Anatomical characteristics of the affected acetabulum were completely matched. Table 2 illustrates the clinical results of patients. In term of the mean OT，there was statistical significance between the 3DP group (126.6 ± 17.6 min) and CS group (156.0 ± 18.6, *P* = 0.003). Mean IBL was statistical significance between the 3DP(115.0 ± 16.9 ml) and CS groups (135.7 ± 26.5 ml, *P* = 0.050), and NORE was statistical significance between the 3DP(3.3 ± 0.8 times) and CS groups (8.6 ± 1.3 times, *P* < 0.001). The follow-up duration of the 3DP and CS groups were 30.3 ± 3.4 and 31.5 ± 2.5 months, respectively. At the last follow-up, the 3DP and CS groups displayed sharp angles of 41.8 ± 2.3° and 42.6 ± 3.1°, respectively; the lateral center-edge angles were 35.6 ± 4.2° and 37.1 ± 2.8°, respectively; the acetabular roof angles were 6.9 ± 1.8 and 9.8 ± 2.6, respectively; and the acetabular head indexes were 86.6 ± 4.1 and 84.3 ± 2.8, respectively. There were no statistical differences in the sharp angle, lateral center-edge angle, acetabular roof angle, and acetabular head index between the 3DP and CS groups (*P* = 0.918,0.846,0.643,0.891). Five patients exhibited a positive crossover sign on the postoperative AP pelvic radiographs in the 3DP group, and six patients in the CS group(*P* = 0.841). In addition, six patients exhibited a positive ischial spine sign on the postoperative AP pelvis radiographs in 3DP group, and seven patients in the CS group(*P* = 0.564). According to the McKay criteria, the 3DP group had 10 excellent, 6 good, and 4 general hips, whereas, the CS group had 12 excellent, 4 good, and 2 general hip. There was no statistical difference between the two groups (*P* = 0.698). In the 3DP group, the postoperative Severin’s grading included 13 hips in grade I, 4 in grade II, 3 in grade II, with a satisfactory rate of 85.0% (17/20). Alternately, the 3DP group had 13 excellent, 4 good, and 3 general hip. In addition, in the CS group, the postoperative Severin’s grading included 11 hips in grade I, 5 in grade II, 2 in grade III, with a satisfactory rate of 88.9% (16/18). There was no statistical difference between the two groups (*P* = 0.945, see Table 2). There were no incisional infection, vascular nerve injury, re-dislocation and varus of hips in both groups. All patients showed no postoperative incision infection, no Kirschner wire penetration into the joint, and no obvious epiphyseal plate, and cartilage injury or other complications.

**Table 2:** Surgical parameters and postoperative follow-up indexes between 3DP group and CS group

Parameter	3DP group (*n* = 20)	CS group (*n* = 18)	*P* value
operation time, h	126.6 ± 17.6	156.0 ± 18.6	0.003
blood loss, mL	115.0 ± 16.9	135.7 ± 26.5	0.050
number of radiation exposures, times	3.3 ± 0.8	8.6 ± 1.3	< 0.001
sharp angle(°)	41.8 ± 2.3	42.6 ± 3.1	0.918
center-edge angle(°)	35.6 ± 4.2	37.1 ± 2.8	0.846
acetabular roof angle(°)	6.9 ± 1.8	9.8 ± 2.6	0.643
acetabular head index(°)	86.6 ± 4.1	84.3 ± 2.8	0.891
crossover sign			
positive	5	4	0.841
negative	15	14	
ischial spine sign			
positive	6	7	0.564
negative	14	11	
McKay criteria			
excellent	10	12	0.698
good	6	4	
general	4	2	
poor	0	0	
Severin’s criteria			
I	13	11	0.945
II	4	5	
III	3	2	
IV	0	0	

Interobserver reproducibility of radiographic hip parameters was evaluated by 3 different radiologists using a two-way, mixed, consistency single-measures intraclass correlation coefficient (ICC). ICC values greater than 0.80 indicate excellent reliability, 0.61–0.80 substantial reliability, 0.41–0.60 moderate reliability, 0.21–0.40 fair reliability, and < 0.20 poor reliability. The ICCs showed excellent reliability for measurements of each parameter (see Table 3). It has been shown to have high interobserver and intraobserver reliability among 3 different radiologists.

**Table 3:** ICCs for intraobserver and interobserver reliability of each parameter

Parameter	Interobserver ICC	Intraobserver ICC
sharp angle(°)	0.824	0.801
center-edge angle(°)	0.762	0.893
acetabular roof angle(°)	0.783	0.866
acetabular head index(%)	0.712	0.842
crossover sign	0.625	0.663
ischial spine sign	0.676	0.758

## Discussion

The purpose of the DDH treatment is to restore the normal anatomical structure of the hip joint, prevent or delay the occurrence and development of osteoarthritis, improve hip joint function, and avoid or delay the implementation of hip replacement [1]. Triple osteotomies aim to reorient the entire acetabulum in older children [15]. Surgery to reorient the acetabulum is the most commonly used procedure in children with well-matched relationship between head and acetabulum. Being a type of reorientation osteotomy that combines ischial, pubic, and iliac cuts, the triple osteotomy permits considerable correction of version, lateral coverage, and anterior coverage. Unlike Salter or Pemberton methods, three combined cuts allow for complete freedom of the acetabular fragment. In 1965, Le Coeur [16] originally reported this osteotomy. In addition to a routine iliac osteotomy, this osteotomy using cuts of the pubis and ischium increased mobilization of the acetabular fragment. The main limitation of this triple osteotomy is the sacrospinous ligament, which remains intact and can limit the acetabular fragment. However, the site of Steel osteotomy was away from the acetabulum, as well as the existing attachments of the sacrospinous and sacrotuberous ligaments, thereby restricting to the acetabular rotation even further [17]. Subsequently, Tönnis further modified the osteotomy site, described a juxta-articular triple osteotomy which conducts the ischial osteotomy proximal to the ischial spine to avoid connection to the sacrotuberous and sacrospinous ligaments, thereby increasing the mobility of the acetabular fragment [18]. According to a biomechanical study, Tönnis osteotomy can produce greater alterations to the lateral center-edge angle. Additionally, it can improve stress distribution of the acetabular cartilage [19]. Vukasinovic et al. reported that the peak stress of the acetabulum after triple osteotomy, as measured by the mathematical model, decreases by 55.9%, compared to before surgery [20].

Tönnis triple pelvic osteotomy indicates satisfactory outcomes [20–22], thus preventing occurrence of hip osteoarthritis [23]. However, long-term follow-up revealed that about 32% of patients require total hip arthroplasty [24]. This is partly due to the pathology of the disease itself, and the ability to accurately rotate the acetabulum after osteotomy, which may also influence long-term outcomes. In addition, the correct direction of the pelvis is difficult to accurate determine on the operating table, and the position of the pelvis can also change during operation. The subjective visual errors of surgeons can result in larger deviations between the direction of the acetabulum after rotation and the original physiological angle. Traditional surgery can only rely on experience to control the osteotomy direction of the ilium, pubis, and ischium, as well as the rotation angle of the acetabulum, which not only increases the risk of vascular and nerve injury, but also may lead to the insufficient or excessive rotation angle of the acetabulum, thus resulting in unsatisfactory coverage of the femoral head. Moreover, due to the limitation in exposure of intraoperative incision and the lack of reliable positioning marks, it is difficult for surgeons to accurately determine the direction of the acetabulum without the aid of multiple X-ray irradiation. Only after long-term training and practical operation can the incidence of postoperative complications be gradually reduced. In recent years, with the popularization of 3D printing in orthopaedic surgery, personalized treatment has increasingly attracted extensive attention from orthopedists.

In recent years, with the popularization of 3D printing in orthopaedic surgery, personalized treatment has increasingly become the concern of orthopedists. The severity of acetabular and femoral head lesions varies in DDH patients, and 2D images cannot fully and intuitively reflect the anatomical characteristics of the patients. Nevertheless, based on 3D CT and MRI, orthopedists can utilize 3D printing to construct a 3D acetabular model to formulate a personalized plan for acetabular osteotomy. 3D printing navigational templates have been widely used in various fields such as trauma or orthopaedics. However, there are few reported applications in pediatric orthopedic disorders. Zheng et al. used a navigation template for screw placement in the treatment of pediatric femoral neck fracture to fulfill the accuracy and reliable correction demands [25].

In this study, a navigation template was used to achieve precise control of the entire operation process by one experienced surgeon. Our results demonstrated that this template can significantly reduce operation time, intraoperative blood loss, and numbers of radiation exposures. Hsieh et al. [26] compared 18 patients who received Ganz osteotomy in the 3DP and CS groups, and reported that in the 3DP group, the operation time was shortened by 21 mins, compared to the CS group. Moreover, the average amount of radiation was only 0.6 times, compared to 4.4 times in the CS group. There were no significant differences in intraoperative blood loss, surgical effect, and complications between the two groups. Indeed, the 3DP navigation template group had significantly shorter operation time and less intraoperative blood loss than the CS group. This proves that the navigation-assisted surgery offers a safer approach, with reduced trauma, compared to CS. In order to restore the normal anatomical structure of the affected hip as best as possible, the healthy hip joint was taken as the standard and the affected hip was mirrored against the healthy hip joint. The normal anatomical standard of the affected hip joint was thus reconstructed, then the mirrored healthy hip joint was used as a standard to guide the control of the rotation direction of the affected hip joint. In preoperative simulation, we first mirrored the healthy hip joint to the opposite side, and then overlapped the healthy hip joint on the affected hip to the maximum extent to obtain the degree and direction of the acetabular defect on the affected side. The iliac osteotomy of the affected hip joint simulated triple osteotomy. The direction of the acetabulum was rotated to the maximum extent to overlap with the mirrored healthy acetabulum, and the acetabulum deformity was thus corrected. In terms of the design of the navigation template, obvious bone markers of the iliac spine were selected as fitting surfaces, and two planes determined the sole rotation direction and angle of the acetabulum. The larger the fitting surface in the design, the more accurate the placement of the navigation template during the surgery.

Following the intraoperative matching of the proximal end of the navigation module with the iliac spine, the acetabulum was only required to rotate to fit the distal end of the navigation module prior to the direct insertion of Kirschner wire for fixation. It was convenient to complete the precise rotation and fixation of the acetabulum at the same time. Moreover, there was no need to perform additional soft tissue dissection, which may aggravate the intraoperative injury associated with navigation template placement. In terms of concerns related to the accuracy of the navigation module, Iwana et al. revealed via measurement of 117 hip replacements rake angles in 103 patients using 3D navigation assisted surgery that the preoperative plan and postoperative actual error was 1.2° ± 1.1°, which was accurate enough to meet surgical needs [27].

At the last follow-up, according to the McKay criteria and Severin’s criteria, there was no statistical difference between the two groups in terms of good rate. It may be because our hospital conducts a large number of such operations and has therefore accumulated relatively rich experience. The merit of our proposed navigation template are as follows: (1) acetabular osteotomy position, osteotomy angle, osteotomy direction, and rotation angle can be precisely controlled, thus, achieving the best orthopaedic effect while avoiding injury of essential nerves and vessels; (2) The rotation angle of the acetabulum is designed using preoperative surgical simulation of patient data, so as to avoid outward movement of the rotating acetabulum center, under- or over-correction, thereby reducing incidence of surgical complications; (3) The presented operation is simple and the operation time, intraoperative blood loss, and number of radiation exposures are vastly reduced. (4) It can reduce the difficulty of operation, shorten learning curve of young doctors, and has promotion and application prospects. Nevertheless, Our study represents a series done by one experienced surgeon who had performed more than 100 conventional triple osteotomy and 30 navigation assisted surgery before the initiation of the study. We agree with other authors that there is a steep learning curve for the Tönnis triple osteotomy，but in part because it is applied in few cases a year even in high-volume hospitals. The presence of a learning curve may imply that those less frequent procedures should be carried out by few surgeons per institution, thus increasing their exposure and ensuring improved results. We found that navigation techniques offered a little additional benefit such as operative time and number of radiation exposures. However, we also found no difference with regard to radiographic correction of the deformity, and the functional outcomes between both groups. There is a strong possibility that surgeons who have less experience in triple osteotomy would profit more from the application of this technology.

There were also some limitations in our study. First, the sample size was small that might have led to bias. Second, a retrospective study allows us to show associations but not to make predictions. We did not collect long-term follow-up data to analyze the outcomes of both the groups. In future studies, we will consider these limitations to provide more reliable results on the progression and have better clinical results on navigational template for Tönnis triple osteotomy.

## Conclusions

In summary, although our study support a role for 3D printing technique in Tönnis triple osteotomy, it seems that the current use of this technique is often unnecessary when an experienced surgeon performs the surgery. However, there is a strong possibility that surgeons who have less experience in triple osteotomy would profit more from the application of this technology.

## Data Availability

All data generated or analyzed during this study are included in this published article.

## Abbreviations

CAD: Computer-aided design
3DP: 3D printing
DDH: Developmental dysplasia of the hip
CS: Conventional surgery
SA: Sharp angle
LCEA: Lateral center-edge angle
ARA: Acetabular roof angle
AHI: Acetabular head index
OT: Operation time
IBL: Intraoperative blood loss
NORE: Number of radiation exposure
COS: Crossover sign
ISS: Ischial spine sign
ICC: Intraclass correlation coefficient
